# Sertraline demonstrates fungicidal activity *in vitro* for *Coccidioides immitis*

**DOI:** 10.1080/21501203.2016.1204368

**Published:** 2016-07-06

**Authors:** Simon Paul, Roger B. Mortimer, Marilyn Mitchell

**Affiliations:** a Department of Internal Medicine, UCSF Fresno Medical Education Program, Fresno, CA, USA; b Department of Family and Community Medicine, UCSF Fresno Medical Education Program, Fresno, CA, USA; c Department of Microbiology, Community Regional Medical Center, Fresno, CA, USA

**Keywords:** Sertraline, coccidioidomycosis, meningitis, MIC, fluconazole

## Abstract

Coccidioidomycosis causes substantial morbidity in endemic areas. Disseminated coccidioidomycosis is an AIDS defining condition and treatment often requires lifelong antifungal therapy. Sertraline, a widely used serotonin-reuptake inhibitor anti-depressant, has demonstrated activity against *Candida* and *Cryptococcus* sp. both *in vitro* and *in vivo*. To evaluate if sertraline has activity against *Coccidioides*, the minimal inhibitory concentration (MIC) and minimal fungicidal concentration (MFC) of sertraline for four clinical isolates of *C. immitis* were determined. Sertraline was observed to have an MIC range of 4–8 µg/ml and MFC also of 4–8 µg/ml for *Coccidioides*. These MIC and MFC results for *C. immitis* are similar to those reported for *Cryptococcus* sp. suggesting sertraline may potentially have utility for the treatment of coccidioidomycosis.

## Introduction

*Coccidioides* species are dimorphic fungi prevalent in arid regions of the Western Hemisphere. Infections with *Coccidioides* sp. cause substantial morbidity and mortality in endemic areas (Ampel 2011), and disseminated coccidioidomycosis is an AIDS defining condition in persons with HIV infection. Treatment is recommended for symptomatic infections, and lifelong treatment is often required when the infection disseminates to bone, lymph nodes, or the central nervous system (CNS). Amphotericin B and fluconazole are the standards of care for treatment; the CNS penetration of fluconazole makes this drug the first choice for most CNS infections. However, even prolonged treatment rarely sterilises the CNS. There is a clear need for more effective treatment options to use alone or in combination against disseminated *Coccidioides* infections, especially for drugs that penetrate the blood–brain barrier.

An interesting potential candidate is the selective serotonin-reuptake inhibitor sertraline, widely used as a well-tolerated anti-depressant medication. Antifungal activity of sertraline was first observed clinically with the resolution of vulvovaginal candidiasis in women treated with sertraline for post-menopausal mood disorder (Lass-Florl et al. 2001). Sertraline was again identified as an antifungal drug using *invitro* screens for off-label antifungal activity of medications currently approved for other clinical uses (Zhai et al. 2012; Butts et al. 2013). The mechanism of fungicidal action of sertraline may include both inhibition of intracellular vesicular transport (Rainey et al. 2010) and inhibition of protein translation (Zhai et al. 2012). Interestingly, sertraline and other serotonin reuptake inhibitors are also being actively investigated for both intrinsic antimicrobial activity and combination treatment of antibiotic-resistant bacteria (Ayaz et al. 2015a, 2015b).

Sertraline demonstrates fungicidal activity against various *Candida* strains *in vitro*, and potent fungicidal activity against *Cryptococcus* sp., with minimal inhibitory concentrations (MICs) of 2–6 µg/ml (Zhai et al. 2012). In a murine model of disseminated cryptococcosis, sertraline was as effective as fluconazole in decreasing CNS fungal burden, and worked synergistically with fluconazole to decrease fungal burden in the spleen, kidney and brain (Zhai et al. 2012). Most recently, studies of the addition of sertraline to standard of care treatment for human Cryptococcal meningitis demonstrated decreased fungal burden in the CSF from the addition of sertraline (Rhein et al. 2016).

Given the promising initial results of sertraline as a potentially additive agent for the treatment of cryptococcal meningitis, and the need for improved treatment options for disseminated coccidioidal infections, *invitro* studies were carried out to determine if sertraline has fungicidal activity against *Coccidioides* sp.

## Methods

Laboratory work was carried out in the biosafety level-3 microbiology laboratory of Community Regional Medical Center, a tertiary care hospital located in an endemic region for coccidioidomycosis (Central California). Fungal isolates from four distinct individual’s clinical specimens were identified as *C. immitis* using the Hologic Gen-Probe nucleic-acid-based assay (Hologic, San Diego, CA, USA). These *C. immitis* colonies were then grown on Sabouraud-dextrose agar slants prior to MIC testing. None of the isolates were stored or further subcultured.

The MIC for each of these four clinical isolates was determined for each drug (or combination) following the reference protocol Clinical and Laboratory Standards Institute M27-S4 and M38-A2 (CLSI 2008). *C. immitis* colonies were scraped from the agar slants, suspended in sterile water and vortexed with sterile glass beads to disaggregate the fungal colonies. The suspended *C. immitis* was then further diluted with sterile water to a concentration of 0.5 McFarland units (530 nm wavelength) and then further diluted 1:100 in Roswell Park Memorial Institute (RPMI) culture medium (RPMI-1640 containing L-glutamine without bicarbonate, pH 7.0 with 0.165 MOPS buffer (Lonza, Allendale, NJ, USA)).

Stock solutions of sertraline (Tocris Bioscience, cas no 7959-97-0) and fluconazole (Santa Cruz Biotechnology sc-205698) were made at 5 mg/mL in dimethyl sulfoxide (DMSO) (Corning Cellgro DMSO MT23950CQC), and then diluted to 10× the desired final concentration using RPMI and filtered using a 0.22-micron syringe filter (Whatman Puradisc cellulose acetate 0.2 µM).

To measure MICs, again, following the reference protocol Clinical and Laboratory Standards Institute M27-S4 and M38-A2 (CLSI 2008), a total of 100 µL of the yeast suspension, 100 µL of the 10× drug concentration (sertraline, fluconazole or for control tubes an equivalent concentration of DMSO/RPMI solution without drug) and 800 µL of RPMI were combined. Susceptibility testing was carried out in twofold drug concentration dilutions ranging from 0.125 to 64 µg/mL for both drugs. For the combination sertraline + fluconazole, 100 µL of each drug was added to the 100 µL yeast suspension + 800 µL RPMI. After mixing, tubes were incubated at 35°C in ambient air until growth was visually evident in the control (no drug added) tubes (5 days). The lowest drug concentration showing no visible growth was recorded as the MIC. The minimal fungicidal concentration (MFC) was determined after this 5-day incubation by plating 0.25 mL of each culture on Saboraud-dextrose agar plates. The lowest drug concentration to show <10 colonies growth was recorded as the MFC.

Standard quality control for yeast susceptibility testing was also performed in parallel with *C. immitis* testing, using *Candida krusei* (ATCC 6258) and *Candida parapsilosis* (ATCC 20019) with MIC results obtained for fluconazole within the acceptable MIC range for these quality control organisms.

Drug interaction was defined based on the fractional inhibitory concentration (FIC): FIC = (MIC drug A in combination/MIC drug A alone) + (MIC drug B in combination/MIC Drug B alone). A drug combination with an FIC ≤0.5 was considered synergistic, FIC >0.5 and ≤1.0 additive, and FIC >1 and ≤2 to be indifferent.

## Results

A single measurement of the MIC and MFC was carried out for each of the four clinical *C. immitis* isolates. The MIC of fluconazole for these *C. immitis* isolates ranged from 4 to 32 µg/mL and for sertraline from 4 to 8 µg/mL (see Table 1). The combination for fluconazole + sertraline showed an MIC range of 2–4 µg/mL of each drug, with an FIC of 0.81 ± 0.78 (±95% confidence interval). The MFC on day 5 of fluconazole for *C. immitis* ranged from 4 to 32 µg/mL and for sertraline from 4 to 8 µg/mL (see Table 1). The combination for fluconazole + sertraline showed an MFC range of 2–4 µg/mL of each drug.

**Table 1.** MIC and MFC results for four clinical isolates of *C. immitis*.

Drug	Isolate			
	1	2	3	4
**MIC (µg/mL)**				
Fluconazole	4	8	16	32
Sertraline	4	4	8	8
Fluconazole + sertraline	4	2	4	4
**MFC (µg/mL)**				
Fluconazole	4	16	16	32
Sertraline	4	8	8	8
Fluconazole + sertraline	2	4	4	4

## Discussion

Sertraline demonstrated MICs for *C. immitis* equal to or lower than the standard of care treatment fluconazole. In combination, the drugs’ effectiveness appears additive. The MFC of sertraline on day five was also equal to or lower than that of fluconazole. Higher MFC values for fluconazole for *Coccidoides* have been previously reported (52 µg/mL) (Ramani and Chaturvedi 2007); our lower MFC values may be due to longer duration of incubation with drug prior to MFC determination. In fact, at shorter time points (12–24 hours), fluconazole demonstrates only static activity against *Cryptococcus* while sertraline demonstrates fungicidal activity (Zhai et al. 2012). It is possible that if earlier time points were studied with *C. immitis*, we would also see fungicidal activity for sertraline vs. only inhibitory activity of fluconazole.

The potential clinical utility of sertraline for treatment of coccidioidomycosis will depend on the drug levels achievable *in vivo* and the tolerability of sertraline (either alone or in combination with other drugs such as fluconazole). While the serum drug level of sertraline at standard approved dosing is significantly lower than the MIC concentrations seen here, sertraline is concentrated in tissues and in the CNS reaches concentrations similar to the MIC for *C. immitis* (Wille et al. 2009; Lewis et al. 2013).

The MIC of sertraline for *C. immitis* is similar to the reported MIC of sertraline for *Cryptococcus neoformans* (Zhai et al. 2012; Rhein et al. 2016). Sertraline has been demonstrated to be effective in mouse models of disseminated cryptococcal infection (Zhai et al. 2012). Initial human clinical trial results of sertraline given in addition to standard of care treatment for cryptococcal meningitis also suggest clinical utility (Rhein et al. 2016), and larger-scale clinical trials are planned. If these trials demonstrate effectiveness and tolerability of sertraline for cryptococcal meningitis, the similar MIC of sertraline for *C. immitis* would suggest sertraline would have potential as an additional agent for the treatment of coccidioidal meningitis and possibly other forms of coccidioidal infection.
